# Effect of PRRSV stability on productive parameters in breeding herds of a swine large integrated group in Spain

**DOI:** 10.1186/s40813-021-00203-4

**Published:** 2021-02-26

**Authors:** D Torrents, J Miranda, PC Gauger, A Ramirez, DCL Linhares

**Affiliations:** 1 Laboratorios Hipra S.A, Av. de la Selva 135, 17170 Amer, Girona, Spain; 2 Veterinary Diagnostic and Production Animal Medicine, Iowa State University College of Veterinary Medicine, 1800 Christensen Drive Ames, 50011-1134 Iowa, USA

**Keywords:** Monitoring, Sows, Farm classification, Weaned piglets, Production parameters

## Abstract

**Background:**

In breeding herds, porcine reproductive and respiratory syndrome (PRRS) clinically manifests as increased abortions, number of stillbirths, and pre-weaning mortality, and as a direct consequence, results in a decrease of the number of piglets weaned per sow per year. Breeding farm classification according the PRRS virus (PRRSV) status (unstable or stable) is a key control strategy for this disease. The aim of this study was to evaluate the production improvement related to achieving a PRRSV stable status in breeding herds in Spain. For this purpose, epidemiological and productivity data were collected from a systematic PRRSV monitoring program in 35 breeding herds from a large integrated swine group in Spain. A comparative statistical analysis was conducted using four key production indicators (KPI) between different PRRSV status and a generalized linear mixed model: weekly abortions/1000 sows (ABTHS), born-alive rate (BAR), pre-weaning mortality rate (PWMR), and number of weaned piglets per 1000 sows (WPTHS).

**Results:**

From the 35 monitored farms during a total period of 58 weeks, we collected 49 to 58 weeks of production data and PRRSV classification status for each study farm. This represented a total of 1997 (741 unstable and 1256 stable) weekly data collected that was eligible for the KPI comparative study. PRRSV stability was associated with significant improvement in BAR (+ 1.10 %, *p* < 0.001), PWMR (-0.88 %, *p* < 0.002) and WPTHS (+ 24.52, *p* < 0.0001).

**Conclusions:**

These results demonstrate for the first time the improved production due to achieving PRRSV stability in breeding herds under field conditions in a European country. Increased number of born-alive piglets and a reduction of piglet pre-weaning mortality represents an increase of 1.28 weaned piglets per sow per year if PRRSV stability was achieved and maintained for one-year period in a breeding farm.

## Background

Porcine reproductive and respiratory syndrome (PRRS) is an endemic swine disease causing significant production and economic losses in pig farms due to reproductive failure in breeding females and respiratory distress in pigs of different ages [1–3]. In breeding herds, PRRSV reproductive disease manifests as an increase in abortions, number of stillbirths, and pre-weaning mortality, and as a direct consequence, results in a decreased number of weaned piglets (WP) per sow per year.

PRRSV epidemiological knowledge at the farm or regional level and farm classification according the PRRS virus (PRRSV) status are a key control strategy of this disease [4, 5]. In 2010, the American Association of Swine Veterinarians (AASV) Board of Directors approved a herd classification system for describing PRRSV status based on determining the shedding and exposure status of the herd [4]. In this classification, four main PRRSV status categories were described: (1) Positive Unstable (PU): Category I; (2) Positive Stable (PS): Category II, (3) Provisional Negative: Category III, and (4) Negative: Category IV, based on the detection of PRRSV by RT-PCR, and PRRSV antibodies by ELISA in serum samples following a standardized sampling protocol.

In breeding herds, PU status is established when breeding herds demonstrate a positive shedding and exposure status. It is also the default category when the herd shedding and exposure status have not been confirmed and testing not conducted. In contrast, PS status is established in breeding herds when there is an absence of clinical signs of PRRS in the breeding herd and lack of a sustained detectable viremia in monthly testing of at least 30 weaning-age pigs for a minimum of 90 days.

Production and economic impact of PRRSV infection in breeding herds have been broadly estimated on the bases of impaired production related to clinical outbreaks in North America [6–8], Europe [9] and Asia [10]. However, association between PRRSV status of breeding herds and their productive performance have been reported just in the United States [11] but not in Europe or Asia. Estimation of the possible enhancement of productive parameters related to the achievement of PS status can provide key information in order to evaluate the real production impact related to PRRSV endemic circulation in positive farms and to consider the cost-benefit of the prevention and control strategies in order to achieve this under field conditions.

The aim of this study was to evaluate the production improvement associated with establishing a PS status in breeding herds under field conditions in Spain. For this purpose, epidemiological and productivity data were collected from a PRRSV systematic monitoring program carried out in breeding herds of a large integrated swine group in Spain. In this monitoring program, breeding herds were classified according to the AASV standardized PRRSV status categories. In addition, weekly abortion rate, percentage of born-alive piglets, pre-weaning mortality rate and number of WP were summarized and reported for each weekly PRRSV status. The major outcome of interest was the change in production between farms in the PU and PS status.

## Materials and methods

### Study design and study population

Data was collected for one-year using a PRRSV systematic monitoring program implemented in 35 Spanish breeding herds between February 2017 and March 2018.

All 35 breeding herds (76,800 sows) belonged to one large integrated group located in Spain. Farm size ranged from 550 to 3900 sows and all farms were considered endemic for PRRSV, *Mycoplasma hyopneumonia*e, *Actinobacillus pleuropneumoniae* and Influenza A virus at the beginning of the study period based on historical health monitoring data of each farm. All farms were located in North-East Spain covering 3 autonomous regions: Navarra (3 farms), Aragon (25 farms) and Catalunya (7 farms). There were four different swine genetics used in the system. Additional individual farm information is summarized in Table 1.

**Table 1:** Demographic information of the farms included in the study

Farm identification	Location (Spanish region)	Sows	Genetic code	Production system^a^
1	Catalunya	3000	A	S1
2	Aragon	1200	A	FTF
3	Catalunya	550	A	FTF
4	Catalunya	3000	A	S1
5	Catalunya	1000	A	S1 + S2
6	Aragon	750	A	S1 + S2
7	Catalunya	3500	A	S1
8	Catalunya	1100	A	S1
9	Aragon	550	A	S1
10	Aragon	1080	A	S1
11	Aragon	800	A	S1
12	Aragon	550	A	S1
13	Aragon	2800	B	S1
14	Aragon	2580	A	S1
15	Aragon	3000	B	S1
16	Aragon	3000	B	S1
17	Aragon	3500	B	S1
18	Aragon	2300	C	S1 + S2
19	Aragon	2400	C	S1 + S2
20	Aragon	2800	B	S1
21	Aragon	1200	A	S1
22	Catalunya	3500	A	S1
23	Aragon	2400	C	S1
24	Aragon	3300	B	S1
25	Aragon	2600	B	S1
26	Navarra	2900	C	S1 + S2
27	Navarra	2900	C	S1 + S2
28	Aragon	3300	C	S1
29	Aragon	620	C	S1
30	Aragon	2800	D	S1 + S2
31	Aragon	950	C	S1 + S2
32	Aragon	3900	B	S1
33	Aragon	3500	B	S1
34	Navarra	2000	B	S1
35	Aragon	1500	B	S1

^a^Production system: S1: Breeding farm; S1 + S2: Breeding + Nursery farm; FTF: Farrow-to-Finish farm

### Diagnostic monitoring protocol

A systematic monitoring sampling for the classification of PRRSV status was designed based on the AASV guidelines [4]. More specifically, blood samples were collected from 30 due-to-wean piglets per farm and month. Sampled piglets were selected according the following criteria: one piglet per litter, preferably low-weight/weak piglets, and preferably from first parity sows. There was a slight modification from the AASV recommended pooling protocol based on common practice in Spain. Serum from individual blood were pooled (5 pools of 6), and tested for PRRSV RNA by RT-PCR [12].

### Molecular diversity of PRRSV

PCR-positive samples were submitted for PRRSV open reading frame 5 (ORF-5) nucleotide sequencing by the Sanger method [13], which allowed differentiating vaccine-like and wild-type PRRSV. PRRSV ORF-5 sequences were analyzed using Geneious 11.1.5 software (Biomatters LTD, NZ).

### Epidemiological data collection

Complementary information related to PRRSV vaccination practices in each farm was collected through an interview of farm staff at every sampling period. This interview included questions about vaccination events performed on the farm before the first sampling time and since the last sampling for later sampling times, recording last vaccination date, and type of vaccine for either sows or piglets. In the case piglets were vaccinated for PRRS around weaning time, samples were collected from the oldest piglets present in farrowing rooms not yet vaccinated.

### PRRSV weekly status classification

PRRSV status classification (PS or PU) was based on the PCR results. It was assumed that all positive farms were PU at the beginning of the study, since no previous systematic PRRSV diagnostics were available. Farms reached positive stable (PS) status after four consecutive negative PRRSV PCR test for all pools tested. PS time was established at the time of the first PCR negative sampling in the series of four negative samplings. When at least one pool was PCR-positive, farms were kept in the PU status. Similarly, farms that reached PS status during the study period returned to PU when at least one pool was PCR-positive. To describe changes in PRRSV status over time, status of the farm was established based on the PCR results of the most recent samples.

When PCR-positive results were obtained from PS farms in samples collected just after a PRRSV mass vaccination of the sows (SMV) with a modified live virus (MLV) vaccine, ORF-5 nucleotide sequence and epidemiologic data were evaluated in order to identify the PRRSV strain. For these cases, PU classification was determined when a PRRSV field strain was identified by ORF-5 nucleotide sequence just after SMV. On the other hand, PS classification was maintained when MLV PRRSV strain was identified as the circulating virus.

This modification of the PRRSV status classification proposed in AASV guidelines [4] was introduced in our study in order to increase the accuracy of classification for these especial cases. Sow mass vaccination was not a common practice at the time and conditions of the proposed scheme. Later, with the increasing use of this practice in sows, they realized that after sows vaccination vaccine virus could be found “accidentally” in offspring piglets for a short period of time in stable farms and so, it could modify temporarily, and under our point of view wrongly, the status classification of the farm.

### Production data collection and KPI calculation

Weekly production data from February 2017 to March 2018 from all 35 farms was provided at the end of the monitoring period in order to calculate the four weekly KPI evaluated in this study: number of abortions per 1000 sows (ABTHS), average number of piglets born alive per litter (BAR), average pre-weaning mortality rate (PWMR), and average number of WP per litter per 1000 sows (WPTHS).

Calculation of weekly KPI is summarized in Table 2.

**Table 2:** Formulas for Key Predictor Indicators to calculate the impact of PRRS on reproductive performance in breeding herds

Key Predictor Indicator (KPI)	Calculation from productive parameters
Abortions x 1000 sows (ABTHS)	(Nº abortions per week / Nº productive sows) x 1000
Born-Alive rate (BAR) (%)	(Nº born-alive piglets per week/ Nº of total born piglets^a^ per week) x 100
Pre-weaning mortality rate (PWMR) (%)	(Nº dead suckling piglets / Nº of total born-alive per week) x 100
Weaned piglets x 1000 sows (WPTHS)	(Nº of weaned piglets per week/ Nº productive sows) x 1000

^a^: total born include still-born, mummified and born-alive

### Statistical analyses

Comparative statistical analysis of weekly KPI between different PRRSV status was performed using a generalized linear mixed model (GLMM) using packages nlme and glmm in R software. In these multivariate models, each KPI was defined as the response variable respectively; meanwhile “PRRSV status” and genetics was defined as the fixed effect explanatory variables. Additionally, “farm” were included as random effect variables in the models in order to control possible variability between farms on KPIs estimations. Goodness of the fit for each model was assessed using Akaike information criterion (AIC).

Parametric or non-parametric analysis was performed according to the data distribution presented for each KPI. Since the variables ABTHS and PWMR did not follow a normal distribution, logarithmic modification of these variables was applied to improve goodness of fit of the models. Moreover, for the ABTHS model, the logarithmic ABTHS variable was treated in the model as a zero-inflated Poisson distribution since it included a large amount of zeros in the dataset of this variable. While for the PWMR model, the logarithmic PWMR variable was treated in the model as a log-normal regression while BAR and WPTHAS were analysed through a Gaussian Process Regression. For statistical analysis significant differences were considered when *p*-value < 0.05.

## Results

From the 35 monitored farms, depending on the time of first and last sampling, we collected between 49 and 58 weeks of production data and PRRSV status classification for each study farm. This represented a total of 1997 weeks of data that were eligible for the KPI comparative study regarding the PRRSV status. According PRRSV status classification, 8 farms remained PU and 10 farms PS for the whole study period, respectively. In contrast, 17 farms demonstrated an alternating PRRSV status during the monitoring period. Altogether, 741 (37.1 %) weeks were classified as PU and 1256 (62.9 %) weeks as PS.

According to complementary information related to PRRSV vaccination practices in each farm collected through an interview of farm’s staff at every sampling period, all farms followed the same vaccination program for sows, based on a 3-times-a-year mass vaccination with a PRRS MLV.

From the primary production data, ABTHS was calculated for all 1997 weeks, BAR for 1991 (99.7 %) weeks, PWMR for 1648 (82.5 %) weeks, and WPTHS for 1985 (99.4 %) weeks. Calculation of BAR, PWMR and WPTHS was not possible for all 1997 weeks due to missing or inaccurate information from some weekly production data. Median and interquartile range (IR) values for each KPI from the whole study group and for each PRRSV status are displayed in Table 3.

**Table 3:** Median and interquartile range (IR) values for each KPI based on PRRSV status

KPI	Overall Median (IR) (*n*= weeks with data)	Positive unstable Median (IR) (*n*= weeks with data)	Positive stable Median (IR) (*n*= weeks with data)
ABTHS	0.694 (0-1.389) (*n* = 1997)	0.680 (0- 1.563) (*n* = 741)	0.633 (0-1.244) (*n* = 1256)
BAR (%)	91.5 (88.4–93.6) (*n* = 1991)	91.1 (87.5–9.37) (*n* = 738)	92.2 (90.0–94.0) (*n* = 1253)
PWMR (%)	12.0 (10.0–15.0) (*n* = 1648)	12.5 (9.4–15.9) (*n* = 609)	12.0 (10.0–14.0) (*n* = 1039)
WPTHS	547.2 (497.1-602.7) (*n* = 1985)	525.4 (457.8–585.0 (*n* = 732)	547.1 (501.5-602.2) (*n* = 1253)

Using GLMM, we observed significant differences for all KPI, except for ABTHS, between.

PU and PS status. Weeks under PS status had a significant increase of BAR (+ 1.10 %) and WPTHS (+ 24.52) and a significant reduction of PWMR (-0.88 %) when compared to PU herd-weeks (Table 4).

**Table 4:** Weekly estimated KPI geometric means and 95 % confidence interval (95 % CI) for Positive unstable and Positive stable

KPI	Positive unstable (Mean (95 % CI))	Positive stable (Mean (95 % CI))	Mean Difference (PS-PU)	*p*-value
ABTHS	0.78 (0.59–1.02) (*n* = 741)	0.84 (0.65–1.09) (*n* = 1256)	+ 0.07	0.297
BAR (%)	90.53 (89.00–92.06) (*n* = 738)	91.61 (90.10–93.12) (*n* = 1253)	+ 1.08	< 0.0001
PWMR (%)	12.15(11.05–13.36) (*n* = 609)	11.20 (10.22–12.28) (*n* = 1039)	-0.95	0.002
WPTHS	507.74 (465.43–550.04) (*n* = 732)	532.95 (491.03–574,88) (*n* = 1253)	+ 25.22	< 0.0001

According to the model used, the genetic was studied as a fix effect and we estimated that genetic D had a significant increase on WPTHS (+ 84.24 weaned piglets/1,000 sows/week, *p* < 0.001) and genetic B significant reduction on WPTHS (-92.05 weaned piglets/1,000 sows/week, *p* < 0.03)

## Discussion

This study presents for the first time comparative field production data from breeding herds regarding their PRRSV status in a European country. Based on the four weekly KPI evaluated in this study, and using a GLMM for its estimation, achieving PRRSV stability can improve productivity in breeding herds. This improvement resulted in 1.10 % increase in piglets born-alive and a 0.88 % decrease in piglet mortality during the 3- weeks lactation period resulting in an overall increase in the number of WP per sow. We estimated an increase of 24.52 WP/1000 sows for each week of PRRSV stability achieved. Therefore, on a yearly bases (52 weeks) we estimate an increase of 1.23 piglets/sow/year (PSY) if PRRSV stability is achieved and maintained for one year.

 The effect of the genetics on WPTHS observed in our study agrees with the reproductive performance of each breed as genetic D is a hyper prolific breed and genetic B is for GP farms to produce F1 sows. On the other hand, genetics didn’t have any impact on the others KPI (BAR, PWMR and ABTH). This lack of effect of genetics on these parameters can be explained since all of them relay rather on the health status and management of the farm than of the reproductive potential of the genetics.

These results partially agree with data reported in two previous studies in the USA, where PRRSV production impacts in breeding herds were estimated to range between 1.44 PSY [11] and 1.92 PSY [8]. Disparity between estimates of PSY regarding PRRSV status could be due to differences in the models used for estimations in each study, differences between pathogenesis of the specific PRRSV infecting the farms [14], differences in biosecurity and management strategies for PRRS control, differences in the immunity and genetic susceptibility against PRRSV in breeding herds [11, 15] or genetics. For this last point, all farms involved in our study were applying three mass vaccinations per year for all sows in addition to occasional mass vaccinations due to new PRRSV clinical outbreaks. The consistency and magnitude of the immune response of the breeding herds in our study, due to this vaccination regime [16–18], could explain the lower impact of PRRSV on the PSY compared to the other two studies where SMV was not implemented at the same regime level.

Although abortions during the late stage of pregnancy is one of the main clinical signs of PRRSV infection in breeding females [19–21], abortion rates were not significantly different between PU and PS weeks in our study. This lack of difference in abortion incidence could be due to the presence of other infectious and non-infectious causes producing abortions at different pregnancy stages. Unfortunately, data reported just from farms just included the total amount of weekly abortion without reporting the possible causes and the stage of pregnancy. Aside, a possible misclassification of the PRRSV status during the 1–3 weeks just after a clinical PRRS outbreak with increasing abortions as a first clinical sign in PS farms could be also contribute to the observation of no differences in abortion rate regarding PRRSV status. The monitoring program used for this study was designed for the PRRSV status classification of breeding herds and not for reporting PRRS clinical outbreaks. When an outbreak occurred in a PS farm, according the monitoring data and criteria, the shift to PU may have been established 1–3 weeks later, possibly misclassifying the first abortion cases under PS status classification.

In order to correct this delay in other future monitoring programs, a complementary, standardized PRRS outbreak reporting system should be implemented. This may include a complementary specific sampling when initial PRRS clinical signs are recognized and using it as a complementary point for PRRSV status classification or establishing a close follow-up of production parameters indicating a PRRSV clinical infection in order to detect early outbreaks and/or to quantify the production losses attributed to PRRSV infection [22].

This delay of PRRSV status classification would not affect the estimation of the other KPIs of the study since they would not be significantly affected until 2–3 weeks after the PRRSV infection has become established in the breeding females, corresponding to the next sampling time and a shift of PRRSV status according to PCR results observed in that sampling.

Alternative population-based PRRSV monitoring methods have been recently implemented for breeding herds that allow affordable, high volume, and high frequency samplings (weekly/daily), such as piglet processing fluids [23, 24] or family oral fluids at weaning [25]. However, at the start of the monitoring program involved in this study, the criteria to establish PRRSV status for breeding herds using these methods was not well defined. Moreover, castration of piglets in Spain is an unusual practice restricting the availability of processing fluids in most of breeding farms. Therefore, we consider the criteria of four consecutive monthly negative results of 5 pools of 6 samples on 30 due-to-wean piglet individual serum samples the most suitable method for monitoring PRRSV positive breeding herds under PRRSV epidemiological field conditions in Spain.

## Conclusions

Using the criteria of four monthly consecutive negative PCR results on 30 individual due-to-wean piglet’s sera pooled by 6, achieving PRRSV stability in breeding herds did not result in a significant reduction in abortion rates but represented a weekly production enhancement due to the increase in BAR (+ 1.10 %) and a decrease of PWMR (0.88 %). These improvements come with an increase of the number of weekly WPTHS (+ 24.52) that would account for an additional 1.3 PSY when achieving and maintaining the PS status for a whole year. These results provide for the first time an estimation of the productive impact of PRRSV stabilization in breeding herds in Spain. The assessment of the productive improvement of PRRSV stabilization can play a key role in the encouragement of farmers to take action on and invest in the control of this disease in breeding herds.

## Data Availability

The datasets used and/or analyzed during the current study are available from the corresponding author on reasonable request.

## References

[1] Rossow KD, Bautista EM, Goya SM, Molitor TW, Murtaugh MP, Morrison RB, Benfield DA, Collins JE (1994). Experimental porcine reproductive and respiratory syndrome virus infection in one-, four-, and 10-week-old pigs. J of Vet Diag Inv.

[2] Christopher-Hennings J, Nelson EA, Nelson JK, Rossow KD, Shivers JL, Yaeger MJ, Chase CC, Garduno RA, Collins JE, Benfield DA (1998). Identification of porcine reproductive and respiratory syndrome virus in semen and tissues from vasectomized and nonvasectomized boars. Vet Pathol.

[3] Keffaber KK (1989). Reproductive failure of unknown etiology. American Association of Swine Practitioners Newsletter.

[4] Holtkamp D, Polson D, Torremorell M, Morrison R, Classen D, Becton L, Henry S, Rodibaugh MT, Rowland RR, Snelson H, Straw B, Yeske P, Zimmerman J (2011). Terminology for classifying swine herds by porcine reproductive and respiratory syndrome virus status. J Swine Health Prod.

[5] Corzo CA, Mondaca E, Wayne S, Torremorell M, Dee S, Davies P, Morrison RB (2010). Control and elimination of porcine reproductive and respiratory syndrome virus. Virus Res.

[6] Baysinger AK, Dewey CE, Straw BE, Brumm MC, Schmitz J, Doster A, Kelling C (1997). The effect of PRRSV on reproductive parameters in swine herds. J Swine Health Prod.

[7] Linhares DCL, Torremorell M, Cano JP, Morrison RB. Comparison of time to PRRSv-stability and production losses between two exposure programs to control PRRSv in sow herds. Prev Vet Med. 2014;116(1–2):111–9. 10.1016/j.prevetmed.2014.05.010 24931129

[8] Valdes-Donoso P, Alvarez J, Jarvis LS, Morrison RB, Perez AM. Production losses from an endemic animal disease: porcine reproductive and respiratory syndrome (PRRS) in selected Midwest US sow farms. Front Vet Sci. 2018;5:102. 10.3389/fvets.2018.00102 PMC5996871 29922683

[9] Nieuwenhuis N, Duinhof TF, van Nes A. Economic analysis of outbreaks of porcine reproductive and respiratory syndrome virus in nine sow herds. Vet Rec. 2012;170(9):225. 10.1136/vr.100101 22238201

[10] Zhang A, Young JR, Suon S, Ashley K, Windsor PA, Bush RD. Investigating the financial impact of porcine reproductive and respiratory syndrome on smallholder pig farmers in Cambodia. Trop. Anim. Health Prod. 2017; 49(4):791–806. 10.1007/s11250-017-1264-1 28316000

[11] Holtkamp DJ, Kliebenstein JB, Neumann EJ, Zimmerman JJ, Rotto HF, Yoder TK, Wang C, Yeske PE, Mowrer CL, Haley CA (2013). Assessment of the economic impact of porcine reproductive and respiratory syndrome virus on United States pork producers. J Swine Health Prod.

[12] Martínez E, Riera P, Sitjà M, Fang Y, Oliveira S, Maldonado J (2008). Simultaneous detection and genotyping of porcine reproductive and respiratory syndrome virus (PRRSV) by real-time RT-PCR and amplicon melting curve analysis using SYBR Green. Res Vet Sci.

[13] Sanger F, Nicklen S, Coulson AR (1977). DNA sequencing with chain-terminating inhibitors. Proc Natl Acad Sci U S A.

[14] Stadejek T, Larsen LE, Podgórska K, Bøtner A, Botti S, Dolka I, Fabisiak M, Heegaard PMH, Hjulsager CK, Huć T, Kvisgaard LK, Sapierzyński R, Nielsen J. Pathogenicity of three genetically diverse strains of PRRSV Type 1 in specific pathogen free pigs. Vet Microbiol. 2017; 209:13–19. 10.1016/j.vetmic.2017.05.011 PMC7127113 28554869

[15] Vincent AL, Thacker BJ, Halbur PG, Rothschild MF, Thacker EL. An investigation of susceptibility to porcine reproductive and respiratory syndrome virus between two genetically diverse commercial lines of pigs. J Anim Sci. 2006; 84(1):49–57. 10.2527/2006.84149x PMC7110373 16361491

[16] Benson JE, Yaeger MJ, Lager KM (2000). Effect of porcine reproductive and respiratory syndrome virus (PRRSV) exposure dose on fetal infection in vaccinated and nonvaccinated swine. J Swine Health Prod.

[17] Scortti M, Prieto C, Simarro I, Castro JM (2006). Reproductive performance of gilts following vaccination and subsequent heterologous challenge with European strains of porcine reproductive and respiratory syndrome virus. Theriogenology.

[18] Simon-Grife M, Ros M, Acal L, March R, Sitja M, Fenech M. Intradermal vaccination with Unistrain®PRRS in gilts protects against reproductive disease after a heterologous challenge. Proceedings 10th ESPHM, Barcelona (Spain). 2018; IMM-014, p352.

[19] Hopper SA, White ME, Twiddy N. An outbreak of blue-eared pig disease (porcine reproductive and respiratory syndrome) in four pig herds in Great Britain. Vet Rec. 1992;131(7):140–4. 10.1136/vr.131.7.140 1413421

[20] Wensvoort G (1993). Lelystad virus and the porcine epidemic abortion and respiratory syndrome. Vet Res.

[21] Mengeling WL, Lager KM, Vorwald AC (1998). Clinical consequences of exposing pregnant gilts to strains of porcine reproductive and respiratory syndrome (PRRS) virus isolated from field cases of “atypical” PRRS. Am J Vet Res.

[22] Silva GS, Schwartz M, Morrison RB, Linhares DCL. Monitoring breeding herd production data to detect PRRSV outbreaks. Prev Vet Med. 2017; 148:89–93. 10.1016/j.prevetmed.2017.10.012 29157378

[23] Vilalta C, Sanhueza J, Alvarez J, Murray D, Torremorell M, Corzo C, Morrison R. Use of processing fluids and serum samples to characterize porcine reproductive and respiratory syndrome virus dynamics in 3 day-old pigs. Vet Microbiol. 2018; 225:149–156. 10.1016/j.vetmic.2018.09.006 30293648

[24] Lopez WA, Angulo J, Zimmerman JJ, Linhares DCL (2018). Porcine reproductive and respiratory syndrome monitoring in breeding herds using processing fluids. J Swine Health Prod.

[25] Almeida M, Zimmerman JJ, Holtkamp DJ, Rademacher C, Rotto H, Schneider P, Linhares DCL. Comparative evaluation of family oral fluids and piglet sera to detect PRRSV RNA by PCR. 49th AASV Meeting Proceedings, San Diego (CA, USA). 2018; P41, p339.

